# Evaluating Hearing Impairment in Different Histopathological Grades of Oral Submucous Fibrosis: An Audiometric Analysis

**DOI:** 10.7759/cureus.100673

**Published:** 2026-01-03

**Authors:** Reeta Jha, Shweta G Thakkar, Soumendu Bikash Maiti, Janvi Gohil, Deepakshi Sabharwal, Jhanvi Shah

**Affiliations:** 1 Department of Oral Medicine and Radiology, Government Dental College and Hospital, Jamnagar, Jamnagar, IND

**Keywords:** areca nut, conductive hearing loss, eustachian tube dysfunction, histopathological grading, oral submucous fibrosis (osmf), pure tone audiometry (pta), tympanometry

## Abstract

Background

Oral submucous fibrosis (OSMF) is a chronic, progressive, and potentially malignant condition primarily linked to areca nut and masala chewing. The fibrotic process not only affects the oral mucosa but may extend to adjacent structures, including the palatal and paratubal muscles, resulting in Eustachian tube dysfunction and consequent auditory impairment. Most individuals habitually place the quid on the left buccal mucosa due to right-handed dominance, which may explain the greater degree of fibrosis and more frequent auditory changes observed on the left side.

Aim

The present study aimed to analyze the level of hearing impairment in different grades of OSMF patients through audiometric and tympanometric findings in both clinical and histopathological grades of the disease.

Materials and methods

A cross-sectional study was conducted on 60 participants, including clinically and histopathologically confirmed cases of OSMF (Grades I-IV) and healthy controls. All subjects underwent pure tone audiometry (PTA) and tympanometric analysis using a calibrated clinical audiometer. Statistical analysis was performed using SPSS version 17.0 (IBM Corp., Armonk, NY), applying chi-square and unpaired t-tests, with p < 0.05 considered significant.

Results

A statistically significant association (p ≤ 0.05) was observed between the clinical and histopathological grades of OSMF, indicating parallel disease progression. Tympanometric evaluation revealed significant middle ear dysfunction predominantly on the left side, consistent with higher fibrosis among left-sided masala users. Although audiometric differences between grades were not statistically significant, asymmetry between right and left ears was evident.

Conclusion

The findings suggest that progressive fibrosis in OSMF can particularly impair Eustachian tube function, leading to conductive hearing loss. Therefore, it is recommended to perform a routine audiological assessment using PTA and tympanometry for early detection and comprehensive management.

## Introduction

Oral submucous fibrosis (OSMF) is a chronic, insidious, and potentially malignant disorder primarily associated with areca nut consumption [1]. It is characterized by progressive fibrosis of the oral mucosa that restricts mouth opening and causes significant functional and psychosocial limitations [2]. Beyond its established intraoral manifestations, OSMF has been reported to extend its effects to adjacent anatomical regions such as the oropharynx and auditory system, where it may contribute to hearing impairments [3].

Histopathological grading of OSMF is essential for evaluating disease progression and systemic involvement [4]. The classification proposed by Pindborg and Sirsat provides a structured framework to correlate histological changes with clinical outcomes [4]. Extracellular matrix remodeling in OSMF progresses in a stage-specific manner, with increased collagen deposition and altered fibroelasticity contributing to mucosal rigidity [5]. The fibrosis and inflammation involving palatal and paratubal muscles are thought to compromise the patency of the Eustachian tube, resulting in altered middle ear ventilation and conductive hearing deficits [6].

Audiometry is the science of measuring hearing acuity and variations in sound using an electro-acoustic device known as an audiometer. Pure-tone audiometry (PTA) is used to determine the hearing thresholds at different frequencies. As a rule, the frequency range of the hearing test varies within 125-8000 Hz, corresponding to speech frequencies, making it essential for hearing assessment. It measures the softest intensity at which tones of varying frequencies are perceived, thereby distinguishing between conductive, sensorineural, and mixed types of hearing loss [7].

Therefore, an audiological evaluation such as PTA and tympanometry could serve as essential diagnostic modalities for detecting early auditory dysfunction in patients with OSMF [3]. In the context of OSMF, PTA can be particularly useful in identifying conductive hearing impairment that may result from compromised Eustachian tube function due to fibrosis of associated muscles [6]. Tympanometry provides an objective assessment of middle ear function by evaluating tympanic membrane and ossicular mobility in response to air pressure changes [3]. It offers critical insights into middle ear pressure, compliance, and the presence of pathologies such as effusion, negative pressure, or ossicular fixation [3]. In OSMF patients, tympanometric abnormalities may reflect impaired Eustachian tube patency and altered middle ear aeration secondary to fibrosis of paratubal muscles [3].

Though previous studies have employed both PTA and tympanometry to study auditory deficits in OSMF patients, they were not differentiated through various stages of histopathological and clinical grades. Audiometric analysis, when integrated with histopathological and clinical staging, can enhance early detection of hearing loss and guide comprehensive treatment planning. This study was therefore designed to evaluate the prevalence of hearing impairment in OSMF patients and to establish its correlation with histopathological as well as clinical grading, thereby providing insights into the diagnosis. 

## Materials and methods

This current cross-sectional observational study was conducted in the Department of Oral Medicine and Radiology, Government Dental College and Hospital, Jamnagar.

The study was conducted after obtaining approval from the Institutional Ethical Committee of the College (EC/NEW/INST/1896/169/02/2024). All the procedures were explained to the patients, and written consent was taken before the analysis.

Sample size is calculated with the sample size (N) formula:

\[N = \frac{2\left( Z_{\alpha} + Z_{\beta} \right)^{2} \times SD^{2}}{d^{2}}\]

Where, SD = anticipated standard deviation from previous studies = 6, Zα = Value of Z when α = 5% = 1.96, Zβ = Value of Z when β = 20% = 0.84, d = minimum expected mean difference = 3. Substituting the values in the formula, the sample size (N) = 62.72. The total number of samples to be included in the study is rounded to 60, considering loss to follow-up and incomplete compliance.

**Figure 1:**
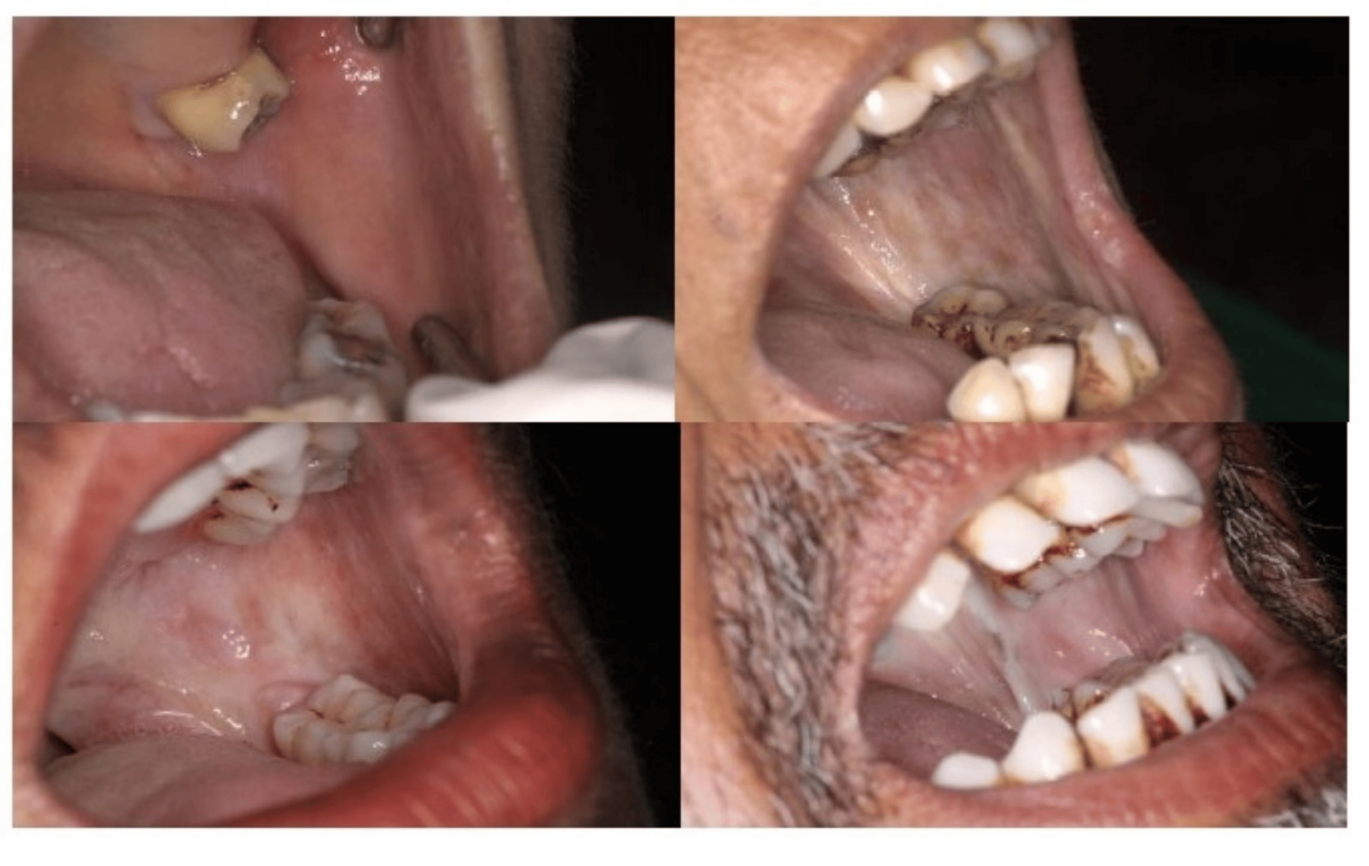
Image showing various clinical grades of OSMF

A total of 60 patients, based on inclusion and exclusion criteria, were included in the study. All the patients were clinically and histopathologically diagnosed with OSMF, with 12 patients each in Grade I, II, III, and IV categories (Figure 1), and 12 healthy individuals without any history of habit with adequate mouth opening were taken as the control. The individual groups had 12 patients for equal distribution of samples among various grades of OSMF. Each group of OSMF Grade with 12 patients was compared with 12 control samples. 

All the patients included were aged between 20-54 years who were clinically and histopathologically diagnosed OSMF patients based on Khanna and Andrade Classification [2,8] and Utsunomiya et al. classification [5], respectively. Those unwilling to participate, those with a history of any other associated oro-facial disorders, surgical intervention involving the oro-facial region, systemic or genetic diseases, pregnancy, or prior middle ear pathology, were excluded from the study. Cases with suspected malignant transformation were also not included in the present study.

Clinical and histopathological evaluation involved detailed case history-taking, including habit history, duration, frequency, and clinical symptoms. Mouth opening was measured using a Vernier caliper, while cheek flexibility and tongue protrusion were also assessed. Clinical staging was documented based on Khanna and Andrade’s criteria [8]. Routine blood investigations (CBC, BT, CT, RBS, HBsAg, HIV) were carried out before biopsy.

A punch biopsy was performed from the most affected/suspected site using the standard procedure. The specimen was preserved in 10% formalin and processed for further histopathological grading.

Audiometric examinations were performed using the Inventis Harp Plus audiometer (Inventis S.r.l., Padova, Italy), capable of both air- and bone-conduction testing. PTA was performed for all OSMF stages and in the control group in a soundproof environment, and tympanometry was done to assess middle ear function. The obtained results were then correlated with the histopathological grading of OSMF.

Audiometric staging followed Goodman’s (1965) scale, in which Grade 1 (10-15 dB) constituted normal hearing; Grade 2 (16-25 dB), minimal hearing loss; Grade 3 (26-40 dB), mild hearing loss; Grade 4 (41-55 dB), moderate hearing loss; Grade 5 (56-70 dB), moderate to severe hearing loss; Grade 6 (71-90 dB) severe hearing loss, Grade 7 (- >90 dB), profound deafness [7].

Tympanometric staging was based on Jerger’s classification [9], wherein Type A (normal) indicates healthy middle ear function; Type As (mild dysfunction), often seen in stiffened middle ear system (otosclerosis, tympanosclerosis); Type Ad (hyper-compliance / laxity), often seen in ossicular chain discontinuity or thin/flaccid TM; Type B (severe pathology), common in middle ear effusion, glue ear, or perforated TM; Type C (early / Eustachian tube dysfunction) indicates negative middle ear pressure, often pre-effusion stage.

For statistical analysis, tympanometry types (A, As, Ad, B, and C) were converted into numerical grades. Therefore, Type A (normal middle ear function) = Grade 1; Type As (mild dysfunction, indicated by stiffened middle ear system, e.g., otosclerosis or tympanosclerosis) = Grade 2; Type Ad (hypercompliance or laxity, e.g., ossicular chain discontinuity or thin/flaccid tympanic membrane) = Grade 3; Type B (severe middle ear pathology, commonly seen in middle ear effusion, glue ear, or tympanic membrane perforation) = Grade 4; and Type C (early/ Eustachian tube dysfunction indicating negative middle ear pressure) = Grade 5.

Ipsilateral reflex can be absent in middle ear pathologies, such as otitis media, ossicular fixation/discontinuity, cochlear pathologies, including moderate to severe sensorineural hearing loss, retrocochlear pathologies, such as acoustic neuroma, auditory neuropathy, and facial nerve abnormalities, including paralysis that affects stapedius muscle innervation.

Statistical analysis

SPSS version 17.0 was used to analyze the descriptive statistics (SPSS Inc., Chicago, IL). The mean ± SD was used to represent continuous variables. Frequencies and percentages were used to express categorical variables. All categorical variables in the study were analyzed using the chi-square test. This included assessing the association between the histopathological grade of OSMF and tympanometric as well as audiometric findings, comparing groups based on clinical load, and evaluating bilateral ear differences for both tympanometry and audiometry. A p-value of <0.05 was considered statistically significant.

## Results

**Figure 2:**
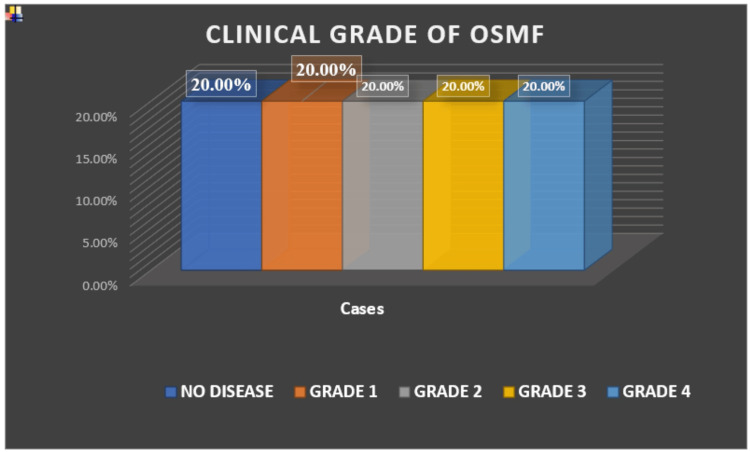
Distribution of study population based on clinical grade of OSMF OSMF: oral submucous fibrosis

A total of 60 patients were included in the study, selected on the basis of inclusion and exclusion criteria. The minimum age observed was 20 years, and the maximum was 54 years. All patients were clinically evaluated and graded according to Khanna and Andrade classification (Figure 2).

The comparison between clinical and histopathological staging yielded significant results, with maximum differences noted in Grade 3 and Grade 4 OSMF. Chi-square test (χ² =3.4) was applied (Table 1).

**Table 1:** Comparison Between Clinical and Histopathological Grades of OSMF A p-value of ≤ 0.05 was considered statistically significant. The p-values presented in the “Total” rows represent the overall chi-square test for the entire table.

Clinical Grade	Histopathological Grade 0	Grade 1	Grade 2	Grade 3	Grade 4	Total	p-value
0	12 (100%)	0	0	0	0	12	
1	0	11	0	1	0	12	
2	0	1	11	0	0	12	
3	0	0	7	5	0	12	
4	0	0	1	6	5	12	
Total	12	12	19	12	5	60	0.001^*^

The audiometric results for both right and left ears showed no statistically significant differences when compared across different clinical and histopathological grades of OSMF (p > 0.05).

The analysis of tympanometric findings revealed a statistically significant association between clinical grades of OSMF in the left ear (p =0.010). Chi-square test (χ² = 10.5 )was applied, which has a significant p-value which is 0.01, indicating progressive middle ear dysfunction with increasing severity of disease. In contrast, the right ear did not demonstrate any significant variation across clinical grades, suggesting that auditory changes in OSMF may present asymmetrically.

**Table 2:** Tympanometric Grades vs Clinical Grades of OSMF Showing Significant Differences in the Left Ear As the findings in the right ear did not show statistically significant differences, a separate table for the right ear was not included. A p-value of ≤ 0.05 was considered statistically significant. The p-values presented in the “Total” rows represent the overall chi-square test for the entire table.

Clinical Grade	Tymp Grade 1	Grade 2	Grade 3	Grade 5	Total	p-value
0	12	0	0	0	12	
1	12	0	0	0	12	
2	12	0	0	0	12	
3	8	2	2	0	12	
4	5	3	2	2	12	
Total	49	5	4	2	60	0.010*

The audiometric evaluation demonstrated a statistically significant difference (p=0.001) based on the chi-square test between the right and left ear comparison, indicating that the degree of hearing loss was not uniformly distributed bilaterally. The left ear showed more pronounced alterations in hearing thresholds across different grades of OSMF, whereas the right ear exhibited comparatively lesser changes (Table 3). Chi-square test (χ² =3.2) was applied, which has a significant p-value (0.001).

**Table 3:** Audiometric grades showing significant bilateral differences A p-value of ≤ 0.05 was considered statistically significant. * significant; ** not significant

Ear	Grade 1	Grade 2	Grade 3	Grade 4	Total	p-value
Left Ear	1 (2%)	47 (94%)	2 (4%)	0 (0%)	50	0.096**
Right Ear	0 (0%)	2 (33.3%)	4 (66.7%)	0 (0%)	6	0.082**
Both	0 (0%)	0 (0%)	0 (0%)	4 (100%)	4	
Total	1	49	6	4	60	0.001*

The tympanometric analysis revealed a statistically significant (p=0.001) bilateral difference, highlighting that middle ear function was affected asymmetrically in patients with OSMF (Table 4). The chi-square test (χ² = 10.3) was applied, and the level of significance is 0.001, which indicates a significant difference bilaterally.

**Table 4:** Tympanometric grades showing significant bilateral differences A p-value of ≤ 0.05 was considered statistically significant. * significant; ** not significant

Ear	Grade 1	Grade 2	Grade 3	Grade 5	Total	p-value
Left Ear	46 (92%)	1 (2%)	3 (6%)	0 (0%)	50	0.010*
Right Ear	1 (20%)	4 (80%)	0 (0%)	0 (0%)	5	0.076**
Both	2 (66.7%)	0 (0%)	1 (33.3%)	0 (0%)	3	
Others	0 (0%)	0 (0%)	0 (0%)	2 (100%)	2	
Total	49	5	4	2	60	0.001*

## Discussion

OSMF is a chronic, insidious, and potentially malignant disorder primarily linked to areca nut consumption [1]. Its clinical impact extends beyond the oral mucosa, with progressive fibrosis compromising oral functions and occasionally extending to the oropharynx and auditory system [2,3,10]. The role of palatal and paratubal muscle fibrosis in compromising Eustachian tube patency has been highlighted as an important pathway leading to middle ear dysfunction and subsequent conductive hearing impairment [4,6].

In the present study, clinical and histopathological grading revealed a significant correlation, reinforcing the understanding that histological alterations parallel the clinical severity of disease [2,5,10]. Clinical and histopathological staging was performed according to Khanna and Andrade’s classification, which provides a structured functional assessment and remains widely used in OSMF research and practice [2,8]. Histopathological grading was carried out using Utsunomiya et al.’s criteria, which offer stage-specific insights into extracellular matrix remodeling and disease progression [5].

Audiometric assessment in our cohort demonstrated minimal to mild hearing loss in the majority of patients, with asymmetry between right and left ears [3,11,12]. While these results did not show statistically significant variation across clinical and histopathological grades, the detection of measurable impairment even in early stages highlights the subtle auditory involvement in OSMF [3,10]. Previous studies, including those by Sreepradha et al. and Devi et al., also identified conductive hearing loss as the most frequent auditory deficit, attributed largely to impaired Eustachian tube function [3,11]. The study conducted by Kumar further confirmed this through a case-control evaluation of auditory tube function in OSMF patients [13].

Notably, other studies, such as those by Desai et al. and Roy et al., reported that advanced stages of OSMF may also show sensorineural or mixed hearing loss, implicating cochlear toxicity as a secondary mechanism [12,14]. Arecoline, the major alkaloid in areca nut, has demonstrated cytotoxic effects on cochlear structures via reactive oxygen species (ROS) generation, supporting a biochemical contribution to hearing loss beyond mechanical obstruction [15]. Findings from oxidative stress studies in OSMF, showing decreased superoxide dismutase and increased lipid peroxidation, further strengthen this association [16].

Our tympanometric findings revealed significant changes in the left ear across higher clinical grades, which can be attributed to most patients having a habit of placing the areca nut on the left side, aligning with previous reports by Shah et al. and Sowbhagya et al., who documented increased abnormal tympanograms with OSMF severity [17,18]. In our study, Type As (Grade 2) and Type C (Grade 5) tympanograms predominated, suggesting reduced tympanic membrane compliance and negative middle ear pressure changes consistent with fibrosis-related impairment of Eustachian tube ventilation [17,18]. Jerger’s classification of tympanometric types provides a robust diagnostic framework and remains a standard in interpreting such findings [9].

The use of audiometric grading according to Goodman’s scale (1965) provided objective stratification of hearing thresholds, ranging from normal to profound impairment [7]. The addition of tympanometric grading allowed us to capture early middle ear changes that may not be apparent in pure-tone thresholds, thereby enhancing diagnostic sensitivity. This dual-modality approach is in line with recommendations from earlier otological research and provides a comprehensive picture of auditory health in OSMF.

Comparison with prior literature reveals both consistencies and divergences. Sreepradha et al. found a linear progression of hearing loss with increasing OSMF severity [3], whereas our findings did not reach statistical significance across histopathological grades. This may be due to differences in methodology, sample size, or demographic variations. Nevertheless, our study supports the notion that OSMF patients, even in early stages, are at risk of subclinical auditory dysfunction and should undergo routine audiological screening.

The clinical implications are clear: OSMF must be approached as a multisystemic disorder requiring multidisciplinary management. Oral physicians are often the first to detect fibrosis-related functional limitations, and recognition of possible auditory involvement should prompt referral to otolaryngologists for further evaluation and management.

Limitations of this study include its single-center design and relatively small sample size. Larger, multicentric, longitudinal studies would better establish the trajectory of auditory impairment in OSMF. Future research should also explore molecular and biochemical mechanisms - including oxidative stress, inflammatory mediators, and extracellular matrix remodeling - to clarify their roles in OSMF-related hearing loss.

## Conclusions

The study establishes a clear association between the severity of OSMF and auditory dysfunction. Significant correlations between clinical and histopathological grades confirm progressive tissue involvement. Tympanometric findings, especially in the left ear, indicate Eustachian tube dysfunction due to fibrosis of palatal and paratubal muscles, likely influenced by habitual areca nut placement on that side. Although most patients showed minimal to mild conductive hearing loss, early detection of such changes highlights the importance of routine audiological evaluation in OSMF. Overall, OSMF should be viewed as a condition with both oral and auditory implications, warranting multidisciplinary assessment and management.
